# Comparative Effect of Rotary Microbrush Combined with Different Chemical Irrigants on Root Dentin Microhardness, Roughness and Bond Strength to an Epoxy-based Sealer

**DOI:** 10.3290/j.ohpd.a44692

**Published:** 2020-07-04

**Authors:** Ana Flávia Simões Barbosa, Larissa Malheiro de Mello, Francine Guedes Teixeira, Fuad Jacob Abi Rached-Júnior, Thais Fantinato Trindade, Walter Raucci-Neto

**Affiliations:** a Graduate Student, Dentistry Department, University of Ribeirão Preto, UNAERP, Ribeirão Preto, SP, Brazil. Contributed significantly to the implementation of the experiment and data processing.; b Undergraduate Student, Dentistry Department, University of Ribeirão Preto, UNAERP, Ribeirão Preto, SP, Brazil. Participated actively in all phases of this work as part of her dissertation.; c Professor, Dentistry Department, University of Ribeirão Preto, UNAERP, Ribeirão Preto, SP, Brazil. Statistical analysis.; d Graduate Student, Dentistry Department, University of Ribeirão Preto, UNAERP, Ribeirão Preto, SP, Brazil. Contributed significantly to the implementation of the experiment and data processing.; e Professor, Dentistry Department, University of Ribeirão Preto, UNAERP, Ribeirão Preto, SP, Brazil. Study design, involved in all phases of this work.

**Keywords:** EDTA, endodontics, epoxy resin based root canal sealer, root canal irrigantsaler

## Abstract

**Purpose:**

This study evaluated the effect of 17% EDTA, 10% citric acid (CA), and 2% chlorhexidine (CHX) activated with a rotary microbrush (CanalBrush) on root dentin microhardness, roughness, and epoxy-based sealer bond strength.

**Materials and Methods:**

One hundred sixty single-rooted bovine incisors were instrumented and divided into 8 groups according to treatment: 1. 17% EDTA; 2. 17% EDTA+2% CHX; 3. 10% CA; 4. 10% CA+2% CHX; 5. 17% EDTA with CanalBrush; 6. 17% EDTA+2% CHX with CanalBrush; 7. 10% CA with CanalBrush; and 8. 10% CA+2% CHX with CanalBrush. Ten roots in each group were split into halves and submitted to microhardness and roughness analyses (n = 10). Following endodontic filling with AH Plus sealer, 10 roots in each group underwent push-out bond strength testing (n = 10). Data were analyzed using two-way ANOVA and Tukey’s tests (α = 0.05).

**Results:**

All groups had similar microhardness values (p > 0.05) which was higher in the apical third than in the middle and cervical thirds (p < 0.05). The CanalBrush groups had higher roughness than the no-activation groups (p < 0.05), with significantly higher roughness in the cervical third than in the apical third (p < 0.05). All groups exhibited similar bond strength (p > 0.05), with the cervical third being higher, followed by the middle and apical thirds (p < 0.05).

**Conclusions:**

Microbrush activation had a direct impact on dentin roughness and did not influence the dentin microhardness or the retention of epoxy-based sealer to the root canal.

Root canal preparation with manual, rotary, or full or partially mechanized rotating (reciprocating) instruments is intended to clean and shape the root canal system. However, this procedure creates a layer composed of organic and inorganic residues, including remnants of pulp tissue, bacteria and their by-products, and dentinal debris.^33^ This smear layer has a negative effect on endodontic treatment success, because it can interfere with the dentin penetration of sealers, resulting in lower retention in the root canal.^31^ An endodontic sealer should have good retention because, under static conditions, it contributes to the minimization or elimination of spaces that could serve as pathways for the infiltration of fluids between the sealer and radicular dentin walls.^28^

Chemicals have been used in endodontic treatment to eliminate bacteria and modify the smear layer composition to facilitate its complete removal. Sodium hypochlorite (NaOCl) has been widely used as a root chemical treatment due to its bactericidal effects and high capacity for organic tissue dissolution.^11,17^ However, due to its limited effects on inorganic materials from the smear layer and its cytotoxicity at higher concentrations, alternative solutions have been developed to complement root canal irrigation. Ethylenediaminetetraacetic acid (EDTA) and citric acid (CA) are used in endodontic treatment as chelating agents, reducing root dentin hardness (to facilitate instrument cutting), and aiding smear layer removal as a complement to the interaction of NaOCl with root canal systems without significantly modifying dentin ultrastructure.^12^

The use of chlorhexidine (CHX) liquid or gel has been proposed to complement the antibacterial effect of NaOCl. Similar antibacterial effects have been demonstrated in liquid or gel presentations. However, the gel form is advantageous due to its lubricating capacity, which increases its clinical use.^10^ Additionally, 2% CHX has been shown to have the same antimicrobial action as that of 5.25% NaOCl^14^ without NaOCl toxicity. However, it has been reported that CHX cannot be advocated as the main standard irrigant in endodontic cases because it is unable to dissolve necrotic tissue remnants and is less effective against Gram-negative than Gram-positive bacteria.^36^ The use of CHX gel as an intracanal auxiliary chemical substance shows antimicrobial action against *Enterococcus faecalis*, and its viscosity provides good mechanical cleaning of the root canal system.^10^ Previous studies have shown that the irrigation of root canals with CHX did not alter^25^ or reduce the bond strength of the sealer to root dentin.^22^

The action of irrigating agents has previously been demonstrated to be enhanced by different agitation strategies, which can improve the contact of these substances with the root canal walls and promote changes in their chemical and physical properties.^3^ During root canal debridement, to reduce the amount of debris within the canal, a flexible microbrush (CanalBrush, Coltène Whaledent; Langenau, Germany) has been suggested. The CanalBrush is available in three sizes (small, medium, and large), which correspond to apical diameters of 25, 30, and 40 µm, respectively, according to the ISO classification.^23^ The manufacturer recommends this brush be used in conjunction with NaOCl at a maximum speed of 650 rpm for up to 30 s and to inspect the brushes for bristle deformation. Debris removal from canal irregularities in the apical part of curved canals is more effective with a canal brush, sonic and ultrasonic irrigation techniques than with syringe irrigation.^23,31^ However, information is lacking on the association of CA and CHX with microbrush agitation.

Therefore, this in vitro study evaluated the effect of the CanalBrush to rub in 17% EDTA and 10% CA (with or without 2% CHX) as a final rinse on dentin microhardness, roughness, and the push-out bond strength of an epoxy-based sealer on the root canal walls. The null hypothesis was that there are no differences in the microhardness, roughness, or push-out bond strength of filling materials using different irrigation protocols.

## Materials and Methods

### Study Design

The variables examined were dentin microhardness, roughness, and sealer retention on the root canal walls. Additionally, the retention of root thirds was compared. Eight experimental groups were formed according to each analyzed variable (n = 10): 1. 17% EDTA; 2. 17% EDTA + 2% CHX; 3. 10% CA; 4. 10% CA + 2% CHX; 5. 17% EDTA with CanalBrush agitation; 6. 17% EDTA + 2% CHX with CanalBrush agitation; 7. 10% CA with CanalBrush agitation, and 8. 10% CA + 2% CHX with CanalBrush agitation.

### Specimen Preparation

Freshly extracted, single-root bovine mandibular anterior teeth were stored in 0.1% thymol for disinfection, and then washed in running water for 24 h. The teeth were examined under a stereomicroscope at 25X magnification (Stemi 2000-C, Zeiss; Jena, Germany) to select those with a similar size and root morphology and verify the absence of cracks and structural defects. A total of 160 specimens were selected (1.27 ± 0.44 mm buccal-lingual root canal diameter and 0.32 ± 0.18 mm mesiodistal root canal diameter) and measured on radiographs. The specimens were transversally sectioned close to the cementoenamel junction with a water-cooled diamond disk (KG Soresen; Barueri, Brazil) in a slow-speed handpiece, to remove the crown and standardize the root lengths at 17 mm. A size 15 K-file (Dentsply-Sirona; Konstanz, Germany) was passively introduced into each root canal to confirm the working length (16 mm). Teeth with laterally displaced foramina and/or a canal length <16 mm were replaced. The cervical third was prepared with an SX instrument (ProTaper-Universal, Dentsply-Sirona), and the complete working length was prepared up to a size 120 K-file (Dentsply-Sirona). The canals were irrigated with 2 ml of 2.5% NaOCl (Farmácia da Terra; Ribeirão Preto, Brazil) at each file change using NaviTip needles coupled to plastic syringes (Ultradent Products; South Jordan, UT, USA). VDW Silver (VDW; Munich, Germany) was used to operate all files.

### Irrigation Protocols

The root apex was closed with wax, and the irrigants were delivered using a precision syringe pump attached to a 30-gauge NaviTip needle inserted into the canal 1 mm from the real working length without damaging the canal walls and aspirated using flexible capillary tips (Ultradent).

- 17% EDTA: The root canal was irrigated with 5 ml of 17% EDTA (Biodinâmica; Ibiporã, Brazil) for 5 min. EDTA was aspirated, and the root was irrigated with 5 ml of distilled water. The total volume of the EDTA solution was 5 ml, and the total exposure time to the EDTA solution was 5 min.^12,22^
- 17% EDTA + 2% CHX: The root canal was irrigated with 5 ml of 17% EDTA for 5 min. EDTA was aspirated, and the root was irrigated with 5 ml of 2% CHX (Biodinâmica; Ibiporã, Brazil) for 3 min. CHX was aspirated, and the root was irrigated with 5 ml distilled water. The total volume of the EDTA solution was 5 ml, and the total exposure time to the EDTA solution was 5 min. The total volume of CHX was 5 ml, and the total exposure time to CHX gel was 3 min.^11^
- 10% CA: The root canal was irrigated with 5 ml of 10% CA for 5 min (Farmácia da Terra; Ribeirão Preto, Brazil). CA was aspirated, and the root was irrigated with distilled water. The total volume of the CA solution was 5 ml, and the total exposure time to the CA solution was 5 min.
- 10% CA + 2% CHX: The root canal was irrigated with 5 ml of 10% CA for 5 min. CA was aspirated, and the root was irrigated with 5 ml of 2% CHX for 3 min. CHX was aspirated, and the root was irrigated with 5 ml distilled water. The total volume of the CA solution was 5 ml, and the total exposure time to the CA solution was 5 min. The total volume of CHX was 5 ml, and the total exposure time to CHX gel was 3 min.
- 17% EDTA with CanalBrush agitation: The root canal was irrigated with 5 ml of 17% EDTA for 4.5 min. EDTA was agitated with a medium-sized rotatory CanalBrush (Coltène; Altstätten, Switzerland) attached to a 600-rpm low-speed handpiece (Dabi Atlante; Ribeirão Preto, Brazil) rotating clockwise. Cervical-apical movements were performed along the root canal walls 2 mm from the working length^23^ for 30 s, during which the irrigating solution was aspirated. EDTA was completely removed, and the root was irrigated with 5 ml distilled water. The total volume of the EDTA solution was 5 ml, and the total exposure time to the EDTA solution was 5 min.
- 10% CA with CanalBrush agitation: The root canal was irrigated with 5 ml of 10% CA for 4.5 min. CA was agitated with the medium-sized rotatory CanalBrush with the same protocol used in group 5. CA was completely aspirated using flexible capillary tips, and the root was irrigated with 5 ml distilled water. The total volume of the CA solution was 5 ml, and the total exposure time to the CA solution was 5 min.
- 17% EDTA + 2% CHX with CanalBrush agitation: The root canal was irrigated with 5 ml of 17% EDTA for 5 min. EDTA was aspirated, and the root was irrigated with 5 ml of 2% CHX for 2.5 min. CHX was agitated with the medium-sized rotating CanalBrush with the same protocol used in group 5. CHX was completely aspirated, and the root was irrigated with 5 ml distilled water. The total volume of the EDTA solution was 5 ml, and the total exposure time to the EDTA solution was 5 min. The total volume of CHX was 5 ml, and the total exposure time to CHX gel was 3 min.
- 10% CA + 2% CHX with CanalBrush agitation: The root canal was irrigated with 5 ml of 10% CA for 5 min. CA was aspirated, and the root was irrigated with 5 ml of 2% CHX for 2.5 min. CHX was agitated with the medium-sized rotating CanalBrush with the same protocol used in group 5. CHX was completely aspirated, and the root was irrigated with 5 ml distilled water. The total volume of the CA solution was 5 ml, and the total exposure time to the CA solution was 5 min. The total volume of CHX was 5 ml, and the total exposure time to CHX gel was 3 min.

### Microhardness and Roughness Analysis

A new CanalBrush was used for each root canal. All brushes were examined under 25X magnification after usage to evaluate bristle deformation.

A total of 10 roots from each group were split into halves. The buccal half was used for the microhardness test, and the lingual half was used for the roughness test. Dentin microhardness and surface roughness were measured following the method described by de Macedo et al.^8^

Under 40X magnification (HMV-2000, Shimadzu; Kyoto, Japan), the Knoop indenter was applied to the dentin surface under a load of 10 g and a dwell time of 15 s. The specimens were individually fixed in the device with the test surfaces perpendicular to the microindenter tip. In each root third, three indentations were made along lines parallel to the edge of the root canal lumen. The first indentation was made 30 μm from the root canal edge, and the two remaining indentations were made at a distance of 30 μm from each other. The representative hardness value for each root third was obtained by calculating the mean of the three indentations.

Surface roughness analysis was performed with a confocal laser-scanning microscope (OLS4000 LEXT, Olympus; Tokyo, Japan). The specimens were fixed on glass slides, while maintaining the root canal surface perpendicular to the microscope objective. For this procedure, a parallelometer and an adhesive material (Pritt, Henkel; Guadalajara, Mexico) were used. The three root canal thirds were scanned with a 20X objective. The microscope had a 405-ηm semiconductor laser that allowed three-dimensional readings of surface roughness (μm^2^). For each scanned root third, a central area of 0.04 mm^2^ was selected where the superficial roughness (S_a_; conforms to ISO 25178) was measured. Representative images of each group were selected for qualitative topographic analysis.

### Root Canal Filling and Push-out Test

Sealer bond strength to the root canal walls was measured following the method described by Macedo et al.^15^

A total of 10 roots from each group were used. The root canals were filled with size 120 gutta-percha master cones (Dentsply-Maillefer; Ballaigues, Switzerland) and L accessory cones (Dentsply-Maillefer) using a lateral compaction technique with AH Plus sealer (Dentsply; Petropolis, Brazil). The gutta-percha cones were cut with a heated instrument and vertically condensed into the canal with a plugger (Dentsply-Maillefer). Sealer excess was removed with cotton pellets. The cervical portions of the root canals were sealed with a quick-setting provisional restorative filling material (Cimpat, Septodont; Barueri, Brazil), and the teeth were immediately stored at 37°C and 95% humidity for a duration three times longer than the regular setting time of the sealer (135 min) prior to the push-out test. The roots were fixed on acrylic plates using wax (Kota-Import; São Paulo, Brazil) and sectioned in a precision cutting machine (Minitom, Struers; Cleveland, OH, USA) at 375 rpm with water cooling. A total of nine 1.5-mm-thick slices were obtained from each root (three per root third) resulting in a total of 720 specimens.

**Fig 1:**
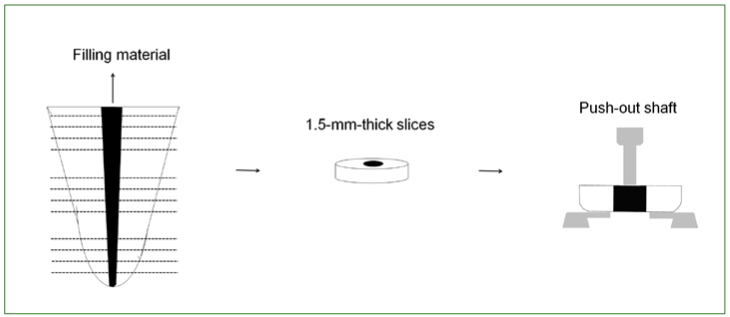
Schematic drawing of the push-out test used for bond strength assessment.

The first slice of each root third was selected, and a stainless steel support was used to hold the specimens in an Instron 3345 universal testing machine (Instron; Canton, MA, USA) with the smaller diameter of the root canal facing upwards and aligned to the shaft that would exert the pressure load on the filling material until debonding occurred. A 6-mm-long shaft with a tip diameter of 0.8, 1.0, and 1.2 mm for the apical, middle, and coronal third, respectively, was used. This method ensured the accurate and reproducible alignment of the specimen, while also maintaining the shaft in a centralized position to prevent it contacting the dentin during testing (Fig 1). The force required to dislodge the filling material (F in kN) was expressed as tension (σ in MPa) by dividing the force by the adhesive area of the filling material (SL in mm^2^) using the following equation: σ = F/A

After the push-out test, the slices were examined with a stereomicroscope (Stemi 2000-C, Zeiss) at 25X magnification to determine the failure pattern. Failure was classified as adhesive when the filling material had totally separated from the dentin (dentin surface with no filling material remnants); cohesive when the fracture occurred within the filling material (dentin surface completely covered by the filling material); and mixed when a mixture of adhesive and cohesive modes (dentin surface partially covered by the filling material) was observed.

### Statistical Analysis

The microhardness, roughness, and push-out data were analyzed by two-way ANOVA (α = 0.001) and Tukey’s tests (α = 0.05) after confirming data homogeneity and normality. All statistical analyses were performed using SigmaStat software, version 3.5 (Systat Software; Chicago, IL, USA). The failure pattern was qualitatively evaluated.

## Results

### Microhardness

ANOVA showed similar microhardness between all irrigation protocols (p > 0.05). The apical third exhibited higher microhardness (p < 0.05) than the middle and cervical thirds, which were similar to each other (p > 0.05). The Knoop microhardness means and standard deviations for all examined groups are shown in Table 1.

**Table 1:** Mean and standard deviations of the microhardness values (Knoop) according to the irrigation protocol and root thirds

Treatments	Root thirds			
	Cervical	Middle	Apical	Total
EDTA	45.18 ± 4.86	47.48 ± 3.41	58.25 ± 5.23	50.14 ± 2.37^A^
EDTA + CHX	43.37 ± 3.69	46.96 ± 3.37	54.90 ± 2.63	48.61 ± 2.38^A^
CA	45.75 ± 4.30	47.82 ± 5.20	56.10 ± 2.55	49.86 ± 2.73^A^
CA + CHX	45.31 ± 3.45	47.20 ± 4.16	53.76 ± 4.54	48.66 ± 1.46^A^
EDTA + CB	43.59 ± 3.78	46.72 ± 4.74	52.72 ± 3.44	47.71 ± 2.56^A^
EDTA + CHX + CB	45.98 ± 3.06	46.33 ± 5.59	54.58 ± 4.57	48.77 ± 1.16^A^
CA + CB	45.55 ± 3.41	47.04 ± 5.53	53.67 ± 6.20	49.61 ± 3.65^A^
CA + CHX + CB	46.37 ± 4.34	46.10 ± 1.80	53.79 ± 5.73	48.91 ± 2.42^A^
Total	45.10 ± 3.86^a^	47.06 ± 4.50^a^	55.12 ± 4.42^b^	

Superscript lowercase letters indicate statistical similarity within columns (p > 0.05), superscript uppercase letters indicate statistical similarity within rows (p > 0.05).

### Roughness

**Fig 2:**
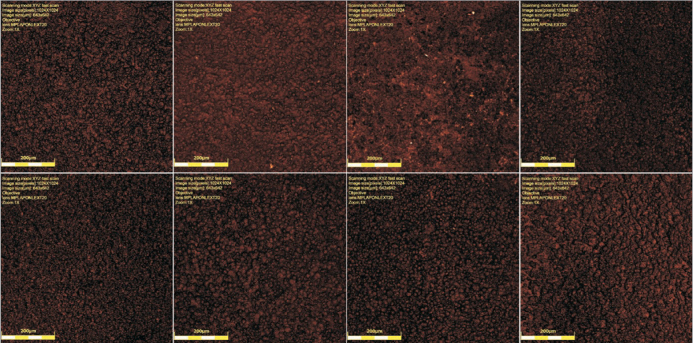
CLSM images of EDTA (A), EDTA + CHX (B), CA (C), CA + CHX (D), EDTA + CB (E), EDTA + CHX + CB (F), CA + CB (G) and CA + CHX + CB (H).

The roughness values of the CanalBrush groups were higher than the other groups (p > 0.05). Significant differences were also observed between the roughness values of root canal thirds among the CanalBrush groups (p = 0.001), ie, the cervical third exhibited higher roughness values than the middle and apical thirds. Representative CLSM images of all groups are shown in Fig 2. The means and standard deviations of the roughness for all examined groups are shown in Table 2.

**Table 2:** Mean and standard deviations of the roughness values (S_a_, μm^2^) according to the irrigation protocol and root thirds

Treatments	Root thirds			
	Cervical	Middle	Apical	Total
EDTA	1.59 ± 0.17	1.63 ± 0.17	1.53 ± 0.18	1.58 ± 0.10A**
EDTA + CHX	1.60 ± 0.25	1.61 ± 0.16	1.60 ± 0.37	1.60 ± 0.11^A^
CA	1.58 ± 0.22	1.60 ± 0.20	1.67 ± 0.24	1.62 ± 0.12^A^
CA + CHX	1.61 ± 0.17	1.54 ± 0.12	1.51 ± 0.19	1.56 ± 0.12^A^
EDTA + CB	2.94 ± 0.11	2.49 ± 0.16	1.84 ± 0.22	2.42 ± 0.10^B^
EDTA + CHX + CB	2.87 ± 0.35	2.49 ± 0.07	1.87 ± 0.27	2.41 ± 0.10^B^
CA + CB	2.97 ± 0.05	2.59 ± 0.48	1.89 ± 0.27	2.49 ± 0.18^B^
CA + CHX + CB	2.74 ± 0.15	2.60 ± 0.38	1.85 ± 0.19	2.39 ± 0.23^B^
Total	2.24 ± 0.69^a^	2.07 ± 0.53^ab^	1.72 ± 0.28^b^	

Superscript lowercase letters indicate statistical similarity within columns (p > 0.05), superscript uppercase letters indicate statistical similarity within rows (p > 0.05).

### Push-out Bond Strength

ANOVA showed similar bond strengths between all irrigation protocols and filling material retention (p > 0.05). Significant differences were also observed in push-out values between root canal thirds (p = 0.001). The cervical third exhibited the highest bond strength, followed by the middle and apical thirds (p < 0.05). The means and standard deviations of the retention values (in MPa) for the displacement of the filling materials during the push-out test are presented in Table 3.

**Table 3:** Means and standard deviations of the push-out bond strengths (MPa) by root third and irrigant protocol

	Root thirds			
Treatments	Cervical	Middle	Apical	Total
EDTA	1.40 ± 0.12	0.77± 0.20	0.47 ± 0.25	0.88 ± 0.06^A^
EDTA + CHX	1.39 ± 0.21	0.79 ± 0.18	0.40 ± 0.18	0.86 ± 0.12^A^
CA	1.26 ± 0.15	0.77 ± 0.13^a^	0.42 ± 0.20	0.82 ± 0.11^A^
CA + CHX	1.36 ± 0.15	0.76 ± 0.24	0.44 ± 0.18	0.85 ± 0.13^A^
EDTA + CB	1.34 ± 0.21	0.88 ± 0.22	0.63 ± 0.15	0.95 ± 0.12^A^
EDTA + CHX + CB	1.27 ± 0.24^a^	0.83 ± 0.17^b^	0.64 ± 0.22^c^	0.92 ± 0.15^A^
CA + CB	1.32 ± 0.23^a^	0.83 ± 0.19^b^	0.65 ± 0.22^c^	0.93 ± 0.10^A^
CA + CHX + CB	1.21 ± 0.29^a^	0.95 ± 0.16^b^	0.68 ± 0.14^c^	0.95 ± 0.11^A^
Total	1.32 ± 0.21^a^	0.83 ± 0.19^b^	0.54 ± 0.22^c^	

Superscript lowercase letters indicate statistical similarity within columns (p > 0.05), superscript uppercase letters indicate statistical similarity within rows (p > 0.05).

Analysis of the mode of failure showed a predominance of cohesive failures in all CanalBrush (agitation) groups. There were many adhesive failures in all non-agitation groups. The distribution of failure modes is presented in Table 4.

**Table 4:** Distribution of failure modes (%) after specimen debonding in each group during the push-out test

Failure pattern (%)			
Treatments	Adhesive	Mixed	Cohesive
EDTA	20	10	70
EDTA + CHX	40	0	60
CA	30	20	50
CA + CHX	40	10	50
EDTA + CB	30	0	70
EDTA + CHX + CB	10	30	60

## Discussion

According to the results of the present study, the null hypothesis was rejected because dentin roughness significantly differed among irrigant protocols. Because agitation with irrigants is intended to enhance the physical and chemical changes in the ultrastructure of the root dentin, higher dentin roughness was expected. However, as the retention of root canal sealers depends on the dentin surface energy,^20^ the increased retention of filling materials is also expected.

The evaluation of dentin microhardness provides an indirect indication of hydroxyapatite content changes in the dental hard tissues, as hydroxyapatite content and tissue hardness are directly correlated.^2^ Therefore, microhardness evaluation makes it possible to evaluate the impact of chemicals on irrigated dentin.^1^ Commonly, chelating agents are used to reduce dentin microhardness to facilitate the preparation of narrow or calcified canals.^34^ Similar to previous studies,^1,4,7,8^ dentin microhardness was measured at depths of 100 μm from the pulp-dentin interface, considering the penetration depth of chemicals irrigants.

The results of the present study support the evidence that EDTA and CA solutions have a marked effect on reducing dentin microhardness due to their chelating properties.^4,7^ However, no additional effect was observed when the EDTA or CA solutions were agitated with the CanalBrush. These results may be associated with the limited action of the CanalBrush with irrigating solutions, because bristle movement within the canal can only disperse the solution.^18^ Different results were observed for ultrasonic or laser agitation, as these methods enhanced the penetration and chelating action of the EDTA solution and promoted an increase in microhardness reduction compared with single file movement within the root.^8,19^

Similar to previous studies,^1,7,8^ it was observed that dentin microhardness of the apical root third was higher than that of the middle and cervical thirds. It has been previously shown that there are structural differences among the root thirds, including a higher tubular density/diameter and collagen fiber density within the cervical third.^29^ This represents an inverse correlation between dentin microhardness and tubular density. Therefore, the mineral content, which is the quantity of hydroxyapatite in the intertubular substance of the apical third, is a considerably influences the intrinsic hardness profile of the dentin structure and the changes in dentin hardness following irrigation treatments.^7^

The effects of root canal irrigants on dentin roughness have been previously evaluated, showing that EDTA ^15,21^ and CA^30^ significantly increase root dentin roughness. In the present study, we observed that EDTA and CA, with or without CHX, had similar dentin roughness. Further, CanalBrush agitation significantly increased dentin roughness compared with that following rinsing with EDTA only or CA only with or without CHX. The use of EDTA ultrasonic or laser agitation has been previously associated with increased dentin roughness because these methods can facilitate the penetration and chelating action of the solution.^15^ However, these results were also associated with increased microhardness reduction. The increased dentin roughness and unchanged dentin hardness observed in the CanalBrush groups may be associated with the effect of bristle rotation and friction on the dentin that was previously chelated with EDTA or CA. Therefore, the CanalBrush movement can only disperse solutions in the root canal and create microgrooves on the root dentin. Corroborating this hypothesis, Mendonça et al^18^ observed that the bristles promote mechanical abrasion of the dentin and hinder the flow of solution, which consequently decreases the removal of debris due to contact with dentinal walls.

Regarding the roughness of root thirds, values were higher in the cervical than in the apical third. These results may be associated with the structural pattern of dentin previously discussed, because the cervical portion of the root canal exhibits higher tubular density/diameter and collagen fiber density, which may be significantly affected by the irrigant protocols, particularly by brush bristles. Thus, an inverse correlation was observed between the roughness and microhardness results because the cervical thirds showed lower microhardness, which could be associated with low mineral content, and higher roughness.

The composition of a sealer and its interaction with dentin can be considerably influenced by dentin ultrastructure modification.^5^ AH Plus sealer, which can be considered the gold standard for testing the resistance of endodontic sealers to dislodgment,^31^ has covalent bonds between the open epoxy rings and the amine groups exposed on collagen, which leads to a strong link between the molecules of the sealer and dentin.^9^ Additionally, high cohesion values between these molecules can improve the bond strength provided by the sealer.^27^ The results of the present study showed that the bond strength of AH Plus sealer was similar among all groups. Therefore, neither the CanalBrush EDTA nor CA agitation influenced the sealer bond strength. A previous study confirm these results, showing increased sealer retention following the ultrasonic^31^ activation of EDTA and similar sealer retention in the CanalBrush and control group (no activation). According to that study, the ultrasonic group had a higher number of open dentinal tubules in the coronal and middle thirds when compared with the CanalBrush group, and the effective removal of the smear layer may improve the adhesion of AH Plus sealer with increased penetration of AH Plus sealer into dentinal tubules. The only factors that contributed to cleaning dentinal tubules and improved sealer penetration were the chemical solutions, which showed similar results.

Regardless of the irrigant protocol, the sealer bond strength decreased from the cervical to the apical root third. According to a meta-regression analysis, the tooth region significantly influences the dislodgement of root filling materials.^6^ The results of the present study are consistent with previous studies,^16,20,31^ which reported that as tubule density decreases from the coronal to apical root third, it could reduce sealer penetration into the tubules of smaller diameter in the apical third. Additionally, the apical third is usually more difficult to access with irrigation solutions, and the consequent incomplete removal of the smear layer may decrease the penetration of sealer into dentinal tubules and thereby affect adhesion in the apical region.^31,35^ Furthermore, the higher collagen fiber density in the cervical third may favor covalent bonding between the open epoxy rings with the amine groups of AH Plus sealer.^13^

In the present study, the mode of failure was mainly cohesive for all groups. These results are consistent with those of previous studies,^24,31^ suggesting that the high adhesion capacity of AH Plus sealer to the root canal dentin may be responsible for this failure pattern.

The use of bovine teeth could represent some limitations to the present study, considering the morphological differences to human dental tissue. However, according to Silva et al,^26^ the possibility to reliably use bovine teeth as a substrate for intratooth modeling would add advantages to the model, such as being able to include tooth samples from animals of the same age and dietary conditions, which may provide dentin of very similar sclerotic patterns, microhardness and elasticity. The authors showed that the use of bovine teeth did not contradict the outcome observed with human samples for the push-out test with different types of sealers including AH Plus, which was used in the present study.

Overall, the present study demonstrated that 17% EDTA and 10% CA agitation with a CanalBrush, with or without 2% CHX, enhanced root dentin roughness, without any additional effect on root dentin microhardness or retention of epoxy-based sealer to the root canal walls, compared with that achieved with irrigants alone. However, further studies are required to assess the effects of the brush activation protocol on the fracture resistance of the root canal and the effects of using different solutions.

## Conclusions

17% EDTA and 10% CA agitation with a CanalBrush, with or without 2% CHX, enhanced root dentin roughness, without any additional effect on root dentin microhardness or retention of epoxy-based sealer to the root canal walls.
